# Whole Slide Image Analysis System for Quantification of Liver Fibrosis

**DOI:** 10.1155/2014/505968

**Published:** 2014-12-15

**Authors:** Tokiya Abe, Yuri Murakami, Masahiro Yamguchi, Yoshiko Yamashita, Tomoharu Kiyuna, Ken Yamazaki, Akinori Hashiguchi, Yutaka Yasui, Masayuki Kurosaki, Namiki Izumi, Michiie Sakamoto

**Affiliations:** ^1^Department of Pathology, Keio University School of Medicine, 35 Shinanomachi, Shinjuku-ku, Tokyo 160-8582, Japan; ^2^Global Scientific Information and Computing Center, Tokyo Institute of Technology, 2-12-1 Ookayama, Meguro-ku, Tokyo 152-8550, Japan; ^3^Medical Solutions Division, NEC Corporation, 7-1, Shiba 5-chome, Minato-ku, Tokyo 108-001, Japan; ^4^Department of Gastroenterology and Hepatology, Musashino Red Cross Hospital, 1-26-1 Kyonan-cho, Musashino, Tokyo 180-8610, Japan

## Background and Aims

Histological evaluation of fibrosis after a liver biopsy is crucial for evaluating the pathology of patients with chronic liver disease. We have reported image analysis allowing quantification of liver fibrosis using Elastica van Gieson (EVG) stained whole slide images (WSIs) of liver biopsy specimens [1]. In this paper, a whole slide image analysis system for quantification of liver fibrosis was developed to apply a large number of cases in routine practice.

## Method

Our system was composed of 2 steps: color correction and tissue classification. Firstly, the color correction was performed by transforming the color distribution from a target WSI into a reference WSI, where the distribution was estimated by two triangle pyramids. Next, the tissue classification was performed by using quadratic discriminant function generated from RGB signal data sets in reference WSIs. After 2 steps, the WSI pixels were classified into five classes corresponding to four tissue areas: collagen fibers, elastic fibers, nucleus, and cytoplasm and one nontissue area (i.e., glass slide). Finally, the area ratios of collagen and elastic fibers were automatically quantified.

## Results

**Figure 1:**
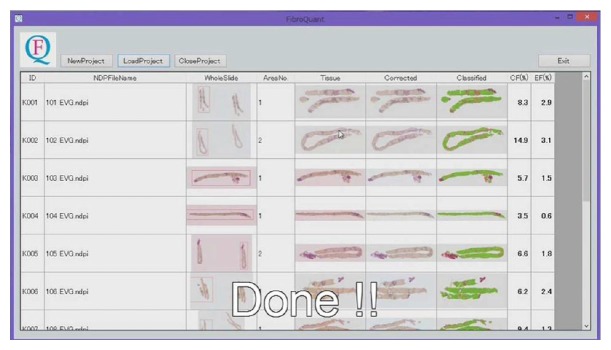
Whole slide image analysis system for quantification of liver fibrosis. The figure shows the color corrected and classified WSI and the area ratios of collagen and elastic fibers obtained by the system.

WSIs of liver biopsy specimens collected from 102 patients with hepatitis C were analyzed by our system [2]. The system successfully corrected the color of any WSI to that of the reference WSI and precisely extracted fine collagen and elastic fibers from portal and periportal areas (Figure 1). The averaged calculation time for WSIs with hundred-millions of pixels solution was about four minutes.

## Conclusions

The whole slide image analysis system could provide quantification of liver fibrosis in biopsy specimens with different color distributions, which can display continuous value, and represent a progression of liver disease.
